# DNA barcoding data release for the Phoridae (Insecta, Diptera) of the Halimun-Salak National Park (Java, Indonesia)

**DOI:** 10.3897/BDJ.11.e104942

**Published:** 2023-07-04

**Authors:** Caroline Chimeno, Stefan Schmidt, Hasmiandy Hamid, Raden Pramesa Narakusumo, Djunijanti Peggie, Michael Balke, Bruno Cancian de Araujo

**Affiliations:** 1 SNSB-Zoologische Staatssammlung München, München, Germany SNSB-Zoologische Staatssammlung München München Germany; 2 Department of Plant Protection, Faculty of Agriculture, Universitas Andalas, Padang, Indonesia Department of Plant Protection, Faculty of Agriculture, Universitas Andalas Padang Indonesia; 3 Museum Zoologicum Bogoriense, Research Center for Biosystematics and Evolution, National Research and Innovation Agency (BRIN), Cibinong, Indonesia Museum Zoologicum Bogoriense, Research Center for Biosystematics and Evolution, National Research and Innovation Agency (BRIN) Cibinong Indonesia; 4 LaBI-UFES, Laboratório de Biodiversidade de Insetos, Universidade Federal do Espírito Santo, Vitória, Brazil LaBI-UFES, Laboratório de Biodiversidade de Insetos, Universidade Federal do Espírito Santo Vitória Brazil

**Keywords:** tropical forest, Indonesia, Brachycera, Phoridae, Malaise trap, biodiversity, DNA barcoding

## Abstract

Launched in 2015, the large-scale initiative Indonesian Biodiversity Discovery and Information System (IndoBioSys) is a multidisciplinary German-Indonesian collaboration with the main goal of establishing a standardised framework for species discovery and all associated steps. One aspect of the project includes the application of DNA barcoding for species identification and biodiversity assessments. In this framework, we conducted a large-scale assessment of the insect fauna of the Mount Halimun-Salak National Park which is one of the largest tropical rain-forest ecosystems left in West Java. In this study, we present the results of processing 5,034 specimens of Phoridae (scuttle flies) via DNA barcoding. Despite limited sequencing success, we obtained more than 500 clusters using different algorithms (RESL, ASAP, SpeciesIdentifier). Moreover, Chao statistics indicated that we drastically undersampled all trap sites, implying that the true diversity of Phoridae is, in fact, much higher. With this data release, we hope to shed some light on the hidden diversity of this megadiverse group of flies.

## Introduction

Indonesia is the world’s largest archipelago, comprising over 17,000 islands and 95,000 kilometres of coastline (Cleary and DeVantier 2011, Cancian de Araujo et al. 2018b). Located off the coast of mainland Southeast Asia in the Indian and Pacific Oceans, it lies across the Equator and links two major biogeographic regions: the Oriental and Australasian (Cleary and DeVantier 2011, Cancian de Araujo et al. 2018a, Kitchener et al. 2004, Indonesia’s tropical climate, geological complexity and extensive territory makes it one of the world’s most biodiverse countries, both for marine and terrestrial organisms and it also harbours high levels of endemism (Cleary and DeVantier 2011). Unfortunately, Indonesia is also renowned for the rate of its biodiversity loss and, as this country has received much less research attention in comparison to others of tropical settings, quantifying this loss is especially difficult (Cleary and DeVantier 2011, Kitchener et al. 2004). Researchers, therefore, find themselves in a race against time to uncover and understand the country's extensive biodiversity before it disappears.

In 2015, the three-year research project, Indonesian Biodiversity Discovery and Information System (IndoBioSys; www.indobiosys.org), was launched to provide a foundation for the large-scale exploration of the species diversity of Indonesia. Funded by the Indonesian and German Ministries of Research and Education, IndoBioSys is a German-Indonesian collaboration between the Museum für Naturkunde Berlin (MfN), the SNSB-Zoologische Staatssammlung München (ZSM) and the Museum Zoologicum Bogoriense, Research Center for Biology – LIPI in Cibinong, (MZB), Indonesia. Its main goal is to develop a standardised framework for species discovery including all associated steps (e.g. processing, documentation, storage, online inventory) (see Schmidt et al. (2017)). One innovative implementation of the project is the use of DNA barcoding methodologies for species identification and molecular-based biodiversity assessments. Another is the development of a comprehensive biodiversity inventory on Barcode of Life Data Systems (BOLD; www.boldsystems.org), an online repository for sequence and metadata.

One work package of IndoBioSys is dedicated to the large-scale assessment of the insect fauna of the Mount Halimun-Salak National Park, which is one of the largest tropical rain-forest ecosystems left in West Java. To achieve this, a total of 34 Malaise traps were set up at four localities of the Park in 2015 and 2016. Malaise traps are commonly used for sampling of terrestrial insects because they provide standardised sampling, are very effective at capturing flying insects and are easy to use (Matthews and Matthews 2017, Schmidt et al. 2019). Previous taxon-specific data releases demonstrate the extreme species-richness of these and other sites that have been sampled in the framework of IndoBioSys (see Cancian de Araujo et al. (2018a), Cancian de Araujo et al. (2018b), Cancian de Araujo et al. (2019), Schmidt et al. (2019), Hilgert et al. (2019)).

Here, we present the results of large-scale DNA barcoding, applied to specimens of Phoridae (scuttle flies) that were captured with the aforementioned Malaise traps. Phorids (scuttle flies) are megadiverse, highly abundant, have a worldwide distribution and occupy all trophic levels in an environment (Disney et al. 2009, Brown and Hartop 2016) making them valuable for biodiversity surveys. Unfortunately, their small size (0.4-5.0 mm) makes them very challenging to study and, worldwide, too little is known about the true diversity of phorids, especially from the Indomalayan region (Brown and Hartop 2016). In this study, we apply biodiversity and ecology statistics to conduct objective and comprehensive evaluations of the diversity of these flies, with the goal of providing a ballpark estimate for species numbers in Indonesia.

## Material and methods

All fieldwork and laboratory procedures were conducted in the framework of IndoBioSys. These are presented in Schmidt et al. (2017), Cancian de Araujo et al. (2018a). Steps that are specific to the analysis of Phoridae are described below.

### Fieldwork and sample processing

**Figure 1.**
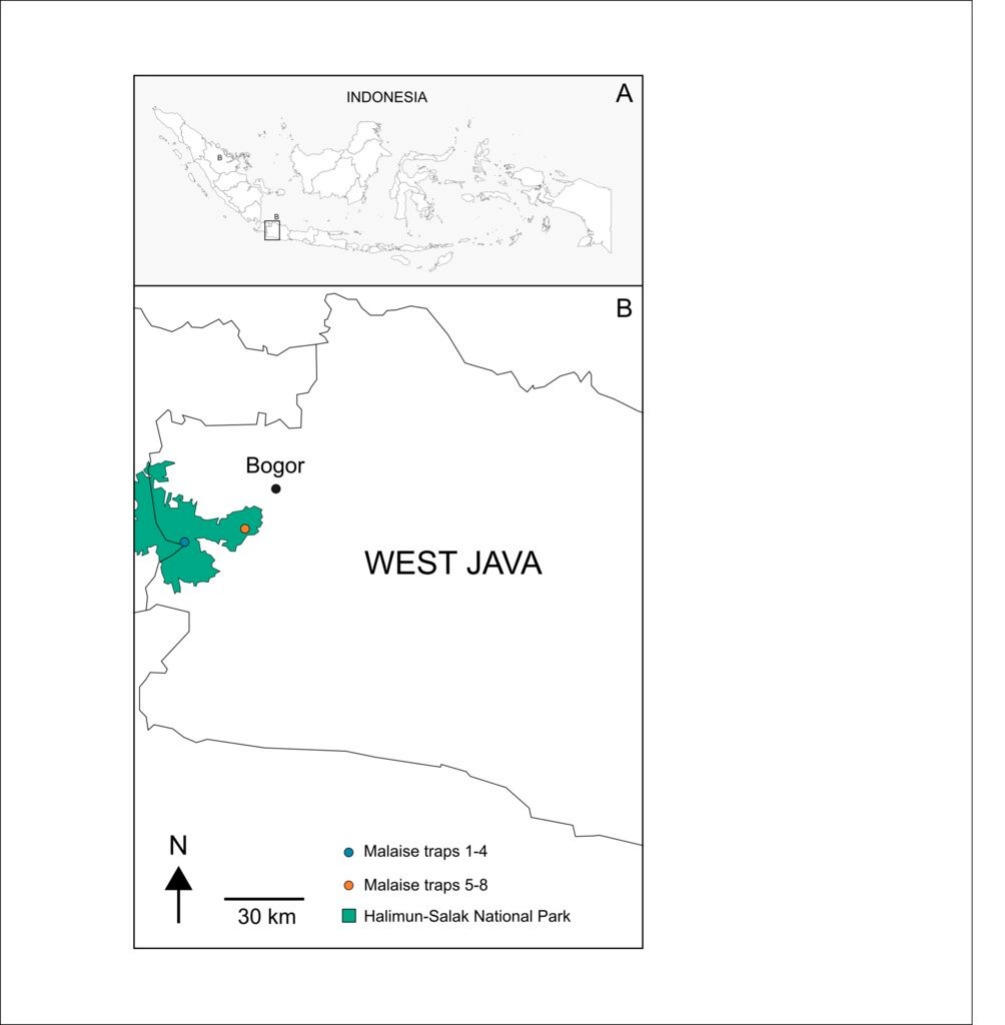
Malaise trap sites in the Halimun-Salak National Park in West Java.

Samples processed in this study originate from eight Malaise traps that were deployed in the Halimun-Salak National Park (Fig. 1, Table 1). These traps were operated from 5 May to 30 July 2016. All collection bottles were replaced fortnightly with new ones containing fresh 96% ethanol. The samples were brought to the laboratory of IndoBioSys located in the MZB in West Java, Indonesia. Here, they were sieved into two fractions using a 3 mm mesh sieve to efficiently separate smaller individuals from larger ones. Specimens of our target taxon, Phoridae, are, therefore, found in the small fractions of samples (< 3 mm). The sample fractions were sent to Singapore for sequencing.

### Laboratory procedures

We extracted the gDNA by submerging the entire specimen in 10 μl of Lucigen QuickExtract solution and heating it to 65°C for 18 minutes and 98°C for 2 minutes. We then amplified the 313 bp fragment of the Cytochrome Oxidase 1 (CO1) gene with the following primer combination: mlCO1intF: 5′-GGWACWGGWTGAACWGTWTAYCCYCC-3′ (Leray et al. 2013) and jgHCO2198: 5′-TANACYTCNGGRTGNCCRAARAAYCA-3′ (Geller et al. 2013). The primers utilised were tagged with 9-bp tags that differed by at least three base pairs. Illumina reads were grouped to each specimen, based on the unique combination of primer tags utilised. We assessed the amplification success rates for each plate through gel electrophoresis for eight random wells per plate and prepared and sequenced a negative control for each 96-well PCR plate.

We pooled all the amplicons into a 50 ml falcon tube, based on the presence and intensity of bands on gels. The pooled samples were cleaned with Bioline SureClean Plus and then outsourced for library preparation at the Genome Institute of Singapore (GIS) using NEBNext DNA Library Preparation Kits (NEB). Paired-end sequencing was carried out on Illumina Hiseq 2500 platforms (2 × 250-bp). We processed the raw Illumina reads through the bioinformatics pipeline and quality-control filters outlined by Meier et al. (2016). We then blasted the resultant sequences to GenBank's nucleotide (nt) database and parsed the BLAST output through readsidentifier (Srivathsan et al. 2015). We then removed barcodes with matches to contaminants at > 97% identity.

### Data analysis

All specimen metadata and sequence data were uploaded to the Barcode of Life Data System (BOLD), an online workbench and database. All data are publicly available on BOLD as a dataset with a citable DOI (dx.doi.org/10.5883/DS-IBSPHOR). We applied the RESL-algorithm that is provided as part of the analysis tools in BOLD, Assemble Species by Automatic Partitioning (ASAP; Puillandre et al. (2021)) and SpeciesIdentifier version 1.9 (Meier et al. 2006) to cluster our sequences at 2 and 3%.

Using R version 4.2.1 (R Core Team 2012), we created accumulation curves of our sequence clusters (via iNEXT; iNEXT package version 2.0.20; Hsieh et al. (2016)) to extrapolate species diversity had we sampled and analysed twice as many specimens and used ChaoSpecies (SpadeR package version 0.1.1; Chao and Jost (2015)) to estimate the species diversity present at the collection sites. Likewise, we created a continuous diversity (via Diversity; SpadeR package) to illustrate the variation in the three standard metrics of biodiversity that are quantified by Hill numbers (q): species richness (q = 0), Shannon diversity (q = 1) and Simpson diversity (q = 2). Hill numbers are a mathematically consolidated group of diversity indices which include relative species abundances in order to quantify biodiversity.

## Data resources

All data are publicly available on BOLD as a dataset with a citable DOI (dx.doi.org/10.5883/DS-IBSPHOR). The R script and input data are deposited on Figshare (R script: https://doi.org/10.6084/m9.figshare.21806370; BIN input data: https://doi.org/10.6084/m9.figshare.21815142; ASAP input data: https://doi.org/10.6084/m9.figshare.21815064).

## Results

**Figure 2.**
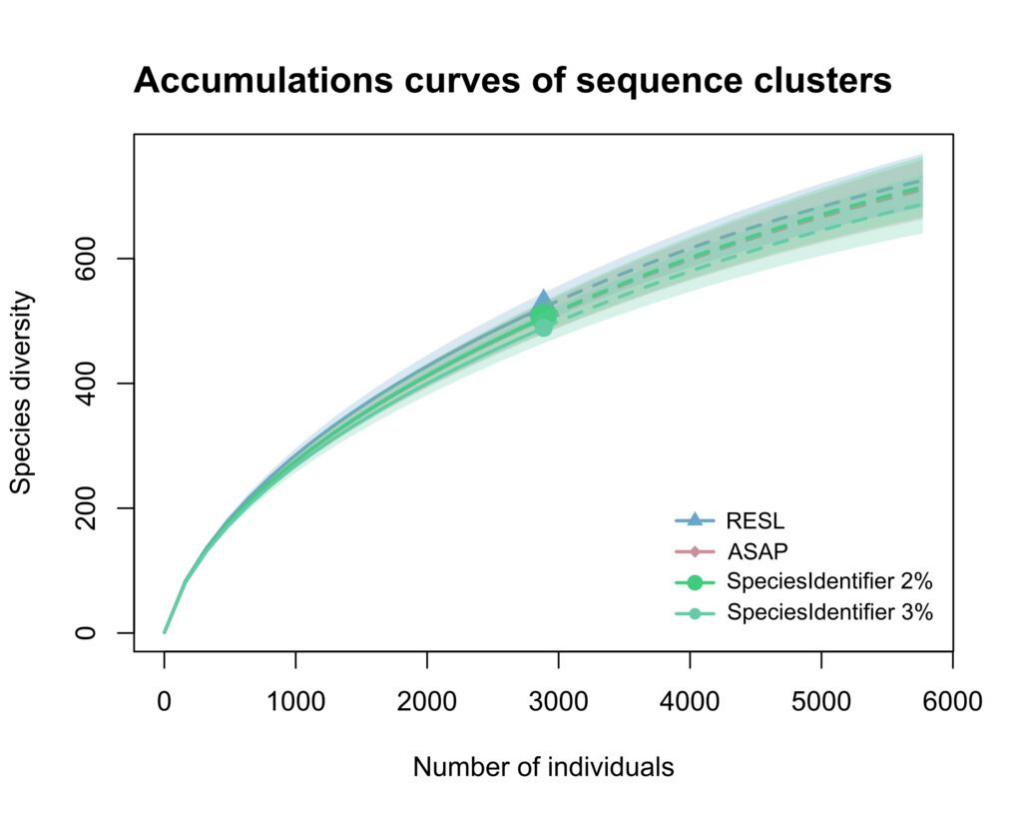
Accumulation curves for numbers of cluster obtained with each clustering method including 95% confidence intervals (RESL, ASAP, SpeciesIdentifier at 2% and 3%).

**Figure 3.**
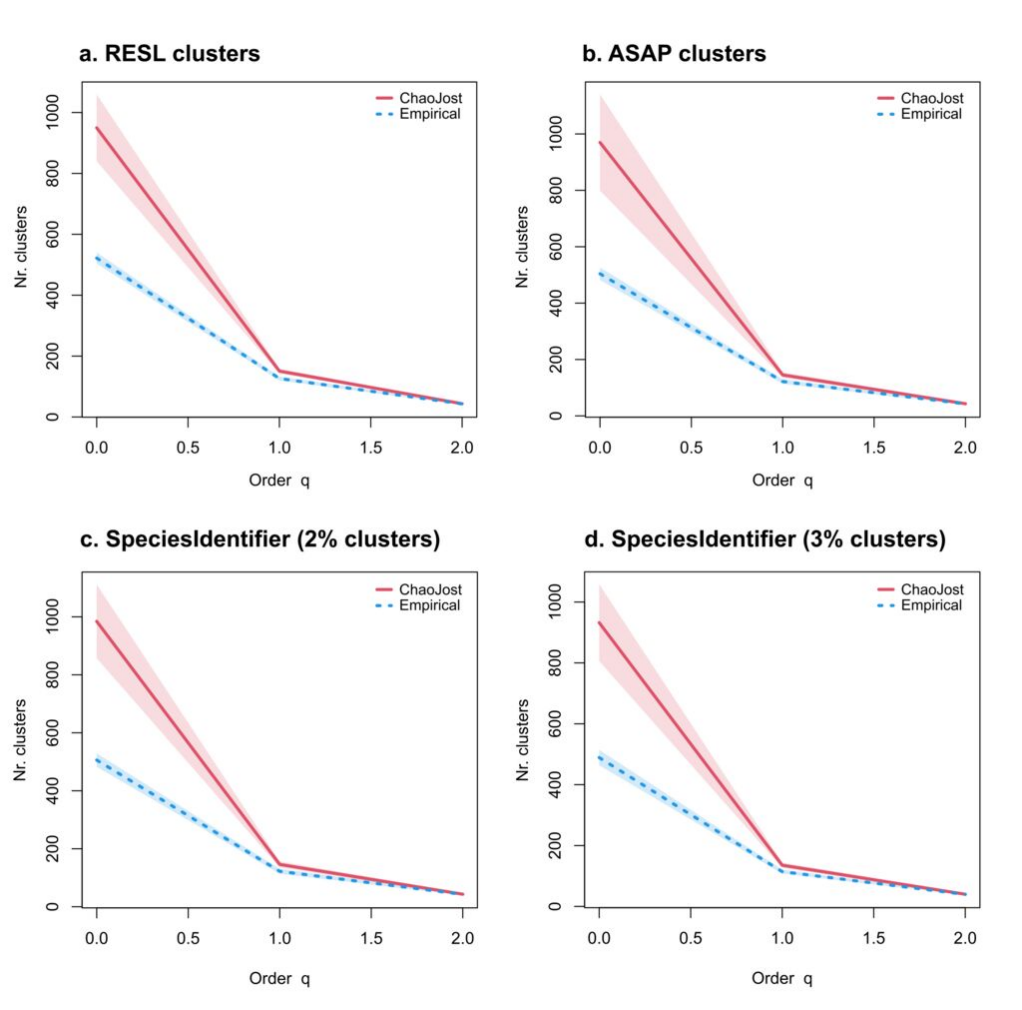
Accumulation curves for number of clusters obtained with each method: a. Diversity profile for sequence clusters obtained with the RESL algorithm; b. Diversity profile for sequence clusters obtained with the ASAP algorithm; c. Diversity profile for sequence clusters obtained with the SpeciesIdentifier using a 2% threshold; d. Diversity profile for sequence clusters obtained with the SpeciesIdentifier using a 3% threshold. The empirical (BIN counts; dotted blue) and estimated (Chao1; red) diversity profiles for communities where Malaise traps were deployed, as quantified by Hill numbers (q) from 0 to 3 with 95% confidence intervals (shaded areas, based on bootstrap analysis of 100 permutations). Species richness is depicted by q = 0; Shannon diversity by q = 1; and Simpson diversity by q = 2.

We processed 5,034 phorid specimens and recovered 2,885 COI-barcode sequences which represents a sequencing success of 57%. We obtained a total of 522 sequence clusters with the RESL algorithm, 504 MOTUs with ASAP and 506 and 489 MOTUs, respectively, when using the 2 and 3% thresholds with SpeciesIdentifier (Table 2). The accumulation curves for each clustering method display identical trends, with overlapping 95% confidence intervals (Fig. 2). This is also visible in the Neighbour-Joining tree (Supplementary Fig. 1), where sequence clustering depicts almost identical results. Applying Chao statistics, we recovered high coverage values of 90% for all datasets. Doubling our sampling effort could have led to an increase in sequence clusters by at least 39% and overall, Chao1 calculations estimate that at least 930 putative species are present in the sampling sites’ communities (Fig. 3). The majority of recovered clusters (70%) are rare, being represented by a single or two specimens only.

## Discussion

Against the backdrop that the majority of the tropic’s biodiversity is associated with insects, studying the megadiverse Diptera in such a setting can be overwhelming. Fortunately, the ongoing development of molecular techniques enables fast and accurate diversity assessments, coupled with a much smaller workload than when applying traditional methodologies. Here, we analyse more than 5,000 phorid specimens that were captured with Malaise traps in the Mt Halimun-Salak National Park located in West Java, Indonesia to provide a first glimpse of their truly impressive species-richness.

As depicted in the diversity profiles (Figs. 2b-c), we significantly undersampled our sampling sites, indicating that the true diversity of Phoridae is, in fact, much higher. This is not surprising, as we: (1) processed samples that were only collected with Malaise traps; (2) processed specimens from only eight samples and (3) have obtained a comparatively low sequencing success. Malaise traps are one of the most effective methods for capturing flying insects (Matthews and Matthews 2017, Schmidt et al. 2019), but as various research has shown, in tropical regions, the highest diversity is found in the canopy of trees (Missa et al. 2009, Basset et al. 2012, Basset et al. 2015). As this diversity is unattainable with Malaise traps, we take it for certain that incorporating more sampling techniques would have substantially recovered more specimens and, in turn, a higher species-richness. Of the few samples that we processed, two did not provide any COI sequences whatsoever. Merging specimens from all samples, we recovered a low total sequencing success of 57%. This was also the case in previous research conducted in the framework of the IndoBioSys project (see Cancian de Araujo et al. 2018a, Cancian de Araujo et al. 2019) and the authors suspect that this was caused by the poor quality of ethanol that was used during collection. This would at least explain why we also obtained low sequencing success despite having applied a completely different laboratory protocol. While specimens in these studies were sent to the Centre for Biodiversity in Genomics for processing and sequencing, our specimens were processed in Singapore. Despite all of these limitations, we obtained more than 500 sequences clusters, which is impressive. Applying three different clustering algorithms (RESL; ASAP; SpeciesIdentifier) provided almost identical results (Table 1; Supplementary Fig.1) and all subsequent biodiversity assessments depicted similar trends with strongly overlapping 95% confidence intervals (Fig. 2). As mentioned, we drastically undersampled the true diversity of Phoridae - Chao1 calculations estimate that almost twice as many putative species are present in the sampled communities.

Scanning through literature, we were only able to find a handful of studies referring to the fauna of Phoridae specifically from the Indomalayan region and those that do only focus on single species or genera without providing information at a larger scale (see Disney 1990, Disney 1986, Zuha et al. 2017, Thevan et al. 2010). Hence, we were not able to obtain any ballpark estimates for phorid species numbers, so in this manner, we are providing a first step towards gauging the diversity of Indomalayan phorid species using a large-scaled data-based approach. We are aware that applying COI-data alone is not ideal for species delimitations. We have uploaded all data to BOLD (making them available to all scientists who wish to conduct further COI-based surveys of Phoridae) and encourage future research to not only use these data, but also implement taxonomic information if possible.

The IndoBioSys project was developed to inventory the insect biodiversity of the Halimun-Salak National Park in order to establish a system that provides baseline information on Indonesia’s entomofauna. With this study (and all past studies conducted in the framework of this project), we show how little is really known about the diversity of generally abundant insect groups like the scuttle flies and that a large proportion of species is still awaiting discovery. For example, when Cancian de Araujo et al. (2018a) and the authors processed 4,531 specimens of Hymenoptera, Coleoptera, Diptera and Lepidoptera from the National Park, they recovered 1,195 species that were completely new to the BOLD database. Another study that conducted canopy fogging in the National Park recovered 747 species of Coleoptera, of which more than half originated from a single tree (see Cancian de Araujo et al. (2019)). Biodiversity in the tropics is patchy and extremely diverse, making it difficult to sample comprehensively. However, with the constant development of large-scale molecular techniques, biodiversity discovery and description are becoming more tangible one step at a time.

## Figures and Tables

**Table 1.** Metadata of the collection samples processed in this study, including Malaise trap data, number of phorid specimens processed with DNA barcoding and number of COI-sequences obtained.

**Collection sample**	**Nr. of phorid specimens**	**Nr. of COI sequences**	**Malaise trap**	**Locality**	**Lat**	**Long**	**Elevation (m a.s.l.)**
1	172	157 (91%)	Trap 1	Cidahu	-6.73761	106.714	1233
2	325	234 (72%)	Trap 2	Cidahu	-6.73438	106.713	1310
3	190	124 (65%)	Trap 3	Cidahu	-6.72846	106.712	1432
4	977	161 (17%)	Trap 4	Cidahu	-6.72636	106.714	1474
5	55	0 (0%)	Trap 5	Cikaniki	-6.75045	106.532	1233
6	258	0 (0%)	Trap 6	Cikaniki	-6.75	106.531	1276
7	1,791	1,465 (82%)	Trap 7	Cikaniki	-6.74863	106.536	1121
8	1,266	744 (59%)	Trap 8	Cikaniki	-6.74775	106.537	1095

**Table 2.** Number of clusters obtained from the COI sequence data of each Malaise trap when applying different clustering algorithms (RESL; ASAP) including output results from biodiversity assessments.

**Output**	**RESL**	**ASAP**	**SpeciesIdentifier (2%)**
Sample size (n)	2,886	2,886	2,886
Number of observed clusters	522	504	506
Number of rare clusters	365	353	354
Sample coverage	90.4%	90.5%	90.5%
Chao1 estimate	950 ± 72	969 ± 80	977 ± 80
Extrapolation to 2n	725 ± 0.9	711 ± 0.9	715 ± 0.9
